# Integrating psychological and cognitive factors in the association between self-reported and objective sleep measures among healthy older adults: a community-based study

**DOI:** 10.3389/fpubh.2025.1735030

**Published:** 2025-12-16

**Authors:** Wei-Yang Lee, Geng-Hao Liu, Ji-Tseng Fang, Ning-Hung Chen, Kuan-Yi Wu, Chih-Ming Lin, Chih-Mao Huang, Tatia M. C. Lee, Shwu-Hua Lee

**Affiliations:** 1 Department of Psychiatry, Chang Gung Memorial Hospital at Linkou, Taoyuan, Taiwan; 2 School of Traditional Chinese Medicine, College of Medicine, Chang Gung University, Taoyuan, Taiwan; 3 Graduate Institute of Traditional Chinese Medicine, School of Traditional Chinese Medicine, College of Medicine, Chang Gung University, Taoyuan, Taiwan; 4 Division of Acupuncture and Moxibustion, Center for Traditional Chinese Medicine, Chang Gung Memorial Hospital at Linkou, Taoyuan, Taiwan; 5 Sleep Center, Chang Gung Memorial Hospital at Taoyuan, Taoyuan, Taiwan; 6 Department of Nephrology, Chang Gung Memorial Hospital at Linkou, Taoyuan, Taiwan; 7 School of Medicine, Chang Gung University, Taoyuan, Taiwan; 8 Department of Pulmonary and Critical Care Medicine, Chang Gung Memorial Hospital at Linkou, Taoyuan, Taiwan; 9 Department of Internal Medicine, Chang Gung Memorial Hospital at Taipei, Taipei, Taiwan; 10 Department of Psychology, The University of Hong Kong, Hong Kong, Hong Kong SAR, China; 11 State Key Laboratory of Brain and Cognitive Sciences, The University of Hong Kong, Hong Kong, Hong Kong SAR, China; 12 Laboratory of Neuropsychology and Human Neuroscience, The University of Hong Kong, Hong Kong, Hong Kong SAR, China

**Keywords:** depression, polysomnography, sleep onset latency, sleep efficiency, wake after sleep onset

## Abstract

**Background:**

Aging disrupts sleep quality, producing fragmented sleep and altered circadian rhythms. Aligning self-reported sleep assessments with objective metrics in older adults is especially important for mental health. This study examined relationships between the Pittsburgh Sleep Quality Index (PSQI), polysomnography (PSG), and psychological outcomes such as distress, loneliness, and cognition to identify objective or emotional-cognitive factors explaining subjective sleep complaints and determine which PSQI subscales best reflected sleep perception.

**Methods:**

In this cross-sectional study, participants aged ≧ 60 years were recruited between September 2019 and October 2020. Each completed the PSQI, underwent PSG, and received assessments of psychological distress, loneliness, and cognition. Spearman correlations tested associations between PSQI subscales and PSG indices. Logistic regression identified influencing indices of poor subjective sleep, and stepwise regression determined which PSQI components were most related to objective and emotional-cognitive indicators, adjusting for demographic and psychological factors.

**Results:**

Data from 89 participants (mean age 73.35 years (± 6.99), 50 women, 56.1%) were analyzed. PSQI subscales correlated with PSG metrics, particularly sleep efficiency, wake after sleep onset, and N2 duration. Regression identified PSG sleep efficiency (SE), sleep onset latency (SoL), wake after sleep onset (WASO), and depression as main influencing indices, with PSQI latency, quality, and disturbance components explaining variance.

**Conclusion:**

PSG SE, SoL, WASO, and depression were dominant influencing indices of subjective poor sleep. Specific PSQI subscales aligned with these indicators, underscoring overlap between subjective and objective measures and the influence of emotional and demographic factors on perceived sleep quality in older adults.

## Background

Sleep structure undergoes marked changes with aging. Older adults typically show reduced total sleep time (TST), sleep efficiency (SE), slow-wave sleep, and rapid eye movement (REM) sleep, along with increased wake after sleep onset (WASO), arousals, and sleep onset latency (SoL) on polysomnography (PSG) (1). These changes are accompanied by altered sleep patterns, with many older adults adopting polyphasic schedules (2). Age-related disturbances are also linked to dysfunction in the suprachiasmatic nucleus of the hypothalamus and to higher rates of primary sleep disorders, including insomnia, sleep-disordered breathing, restless legs syndrome, and circadian rhythm disturbances (3). Although aging causes substantial changes in sleep, it does not always result in greater complaints about sleep quality (4). Older adults often show more pronounced fragmentation on actigraphy but report shorter latency and fewer awakenings in sleep diaries (5).

Studies indicate that in older adults, objective metrics such as TST, SE, and WASO strongly influence subjective sleep disturbances, with prolonged wakefulness at night impairing sleep perception (6, 7). However, discrepancies remain: poor sleepers may overestimate sleep duration and SE, highlighting variability between subjective and objective evaluations (8). Self-reported data from individuals with minor depression also show misalignment with PSG, influenced by psychological factors such as anxiety and depression, which affect daytime alertness and perceived sleep quality (9). Thus, while self-reported sleep data can align with objective measures in some respects, clinical assessment should balance these subjective reports against objective findings to improve diagnosis and treatment (10).

Research has increasingly examined the relationship between sleep quality and mental health, comparing subjective and objective measures of sleep and their effects on psychological well-being. Poor sleep quality has been linked to elevated risk of depressive symptoms, as seen in Korean adults, where interventions targeting sleep behavior have been suggested as preventive strategies against depression (11). Among older adults, a meta-analysis found a strong association between sleep deprivation and depression, underscoring the role of sleep in mental health management for this population (12). Self-reported measures, such as the Pittsburgh Sleep Quality Index (PSQI), show that higher scores—indicating poorer sleep quality—are closely correlated with psychological distress, including anxiety, stress, and depressive symptoms (13). However, although PSQI scores capture perceived complaints, they are less effective in detecting abnormalities measured by PSG, suggesting that the impact of sleep quality on mental health may be more subjective than physiological (8). Collectively, poor self-reported sleep quality exacerbates emotional distress and contributes to compromised mental health.

In summary, self-reported assessments—particularly among individuals with depression and anxiety—often show limited concordance with objective PSG findings. Instruments such as the PSQI effectively capture perceived disturbances but may not reflect underlying physiological abnormalities. Therefore, this study aimed to: (1) examine associations between specific PSQI components and PSG sleep parameters; (2) identify key objective sleep indicators most strongly associated with subjective poor sleep, after sequentially controlling for demographic factors, psychological distress, loneliness, and cognition in healthy older adults; and (3) determine which PSQI subscales best represent subjective sleep perception by examining their links with relevant objective and emotional-cognitive indicators, thereby clarifying the convergence or divergence between subjective and objective sleep and their associations with emotional and cognitive outcomes.

## Methods

### Design and participants

This cross-sectional observational study, titled “Integrating Systematic Data of Geriatric Medicine,” was conducted from September 2019 to October 2020 to identify strategies for promoting healthy aging. Older adults living in the community were recruited from the Songshan District of Taipei City and from Chang Gung Health and Culture Village in Taoyuan City. Participants were recruited through random sampling and screened according to the Health Check-up Process. Inclusion criteria were: (1) age ≥ 60 years; (2) at least one visit to Chang Gung Memorial Hospital within the year before recruitment; and (3) residency in Taiwan for > 180 d during that year. Exclusion criteria were: (1) significant organ-system abnormalities; (2) severe autoimmune disorders; (3) ongoing cancer treatment at recruitment; (4) antibiotic use within the preceding month; (5) Ascertain Dementia 8 score ≥ 2, Mini-Mental State Examination score ≤ 26, or Geriatric Depression Scale score ≥ 5; (6) physician-diagnosed dementia, stroke, Parkinson’s syndrome, Parkinson’s disease dementia, epilepsy, brain tumors, or history of psychotic disease, major depressive disorder, anxiety disorder, bipolar disorder, alcohol or drug abuse, or significant head trauma with loss of consciousness; (7) substantial hearing, vision, or cognitive impairment interfering with clinical assessment; and (8) frailty preventing standing or walking. Only participants with complete PSG assessments were included in the present analysis. This study received approval from the Institutional Review Board of the Chang Gung Medical Foundation, Taiwan (approval number 201900702A3), and it was carried out with the principles of the Declaration of Helsinki and written informed consent from all participants. As a pilot, the study aimed to establish preliminary processes and data collection under resource constraints, and therefore targeted an initial enrollment of 100 participants able to complete the program.

### Measurement

Participants’ demographics, medical histories, polysomnographic indices, and responses to questionnaires on psychological distress, loneliness, and cognitive functioning were recorded. Relevant medical records were also reviewed during interviews.

### PSQI

The PSQI is a self-administered 19-item questionnaire evaluating self-reported sleep quality over the preceding month. Its items generate seven component scores: sleep quality, sleep latency, sleep duration, habitual sleep efficiency, sleep disturbances, use of sleeping medication, and daytime dysfunction. Each component is scored from 0 to 3, and their sum yields a global score ranging from 0 to 21, with higher scores indicating poorer sleep quality. The PSQI demonstrates excellent internal consistency (*α* = 0.83). Its clinical and psychometric properties have been extensively validated, showing a sensitivity of 89.6% and specificity of 86.5% for detecting sleep disorders at a cutoff score of 5 (14).

### PSG

Participants underwent standardized home-based PSG using the portable Philips Alice 6 lDe monitoring system (Philips Healthcare), which records EEG, EOG, chin EMG, airflow, thoracoabdominal respiratory effort, oxygen saturation (SpO₂), body position, and snoring. Sleep stages were scored in 30-s epochs according to the American Academy of Sleep Medicine (AASM) scoring criteria (15). The following PSG metrics were calculated: total sleep time (TST, the duration from sleep onset to final awakening minus wakefulness periods); SE, the percentage of time asleep relative to total time in bed; SoL, the interval from lights off to sleep onset; WASO, time awake after initial sleep; non-REM stages 1, 2, and 3 (percentage of total sleep time); REM sleep (percentage of total sleep time); and the arousal index (average arousals per hour of sleep). Overnight home-based polysomnography (PSG) was performed using a portable PSG system. Installation and equipment setup were completed by a professional PSG technician with the assistance of a project staff member. Scoring was performed by sleep technicians with > 10 years of experience, and final interpretation was conducted by a sleep specialist. Participation in multicenter studies has validated the reliability of our PSG scoring and reporting, ensuring consistency with international best practices (16, 17).

### Psychological distress, loneliness, and cognitive functioning

Each participant’s psychological distress, loneliness, and cognitive functioning were assessed using validated scales addressing different mental domains. The 17-item Hamilton Rating Scale for Depression (HAM-D) evaluates depression by assessing core symptoms, sleep habits, daily functioning, anxiety, and delusions. A score ≤ 7 generally indicates remission (18). The 14-item Hamilton Rating Scale for Anxiety (HAM-A) measures anxiety severity, distinguishing between psychological and physical symptoms, with a score ≤ 7 reflecting no or minimal anxiety (19). The Chinese clinician-administered Apathy Evaluation Scale (AES) includes 18–72 points, with higher scores indicating greater apathy. It shows strong internal consistency (*α* = 0.90) and test–retest reliability (0.86–0.88). A score > 34 typically indicates apathy (20, 21). The triggers of suicidal ideation inventory (TSII) measures factors associated with suicidal ideation in older adults, including emotional distress, family and financial concerns, and self-worth issues. It demonstrates good internal consistency (*α* = 0.78), with a cutoff score of 2 used to detect triggers (22). The revised 20-item Chinese version of the University of California, Los Angeles Loneliness Scale measures perceived loneliness, with total scores ranging from 20 to 80; higher scores indicate greater loneliness (23–25). Finally, the Everyday Cognition Scale (ECog) is a 39-item informant-rated questionnaire designed to assess daily cognitive functioning over the past 10 years. It evaluates six cognitive domains—memory, language, visuospatial abilities, planning, organization, and divided attention—using a 4-point scale, with higher scores indicating greater functional decline (26).

### Statistical analysis

All statistical analyses were conducted using IBM SPSS Statistics for Windows, version 27.0. The Kolmogorov–Smirnov test assessed the normality of continuous data, and results showed the survey data were not normally distributed (*p* < 0.05) (27, 28). Accordingly, Spearman correlation analysis was used to compare the PSQI subscales with PSG indices. Participants were also categorized using a PSQI cutoff score > 5 to indicate poor sleep.

Logistic regression analyses examined associations between subjective poor sleep and objective sleep indicators, psychological distress, loneliness, and cognitive function. Subjective poor sleep status (poor vs. non-poor) was the dependent variable, while objective sleep measures, psychological distress, loneliness, and cognition served as independent variables. Model 1 adjusted for demographic factors (age, sex, and education). Model 2 additionally adjusted for psychological distress and loneliness. Model 3 further incorporated cognitive function, based on Model 2.

Stepwise regression analysis was performed to explore associations between PSQI subscales and indices significantly related to subjective poor sleep in Model 3. Each analysis included the same independent variables: three demographic factors (age, sex, and education level) and the seven PSQI subscales. Demographics were included as covariates to control for potential confounding. Statistical significance was defined as a two-tailed *p*-value < 0.05.

## Results

### Sample characteristics

**Figure 1:**
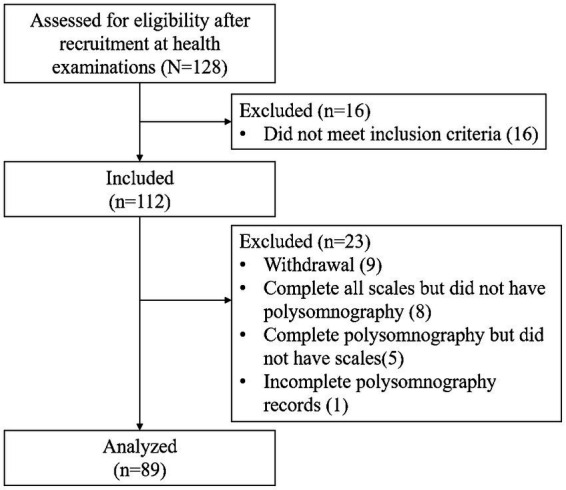
Flow diagram of the inclusion process.

Between September 2019 and October 2020, 128 participants were enrolled in the Integrating Systematic Data of Geriatric Medicine study, which investigated strategies for promoting healthy aging. Sixteen were excluded for not meeting the inclusion criteria. Of the 112 eligible participants, 9 (8.0%) withdrew, 5 (4.5%) did not complete the scales, 8 (7.1%) did not complete the PSG, and 1 (0.9%) had incomplete PSG records, resulting in 23 exclusions. Thus, 89 participants (79.5%) completed both PSG and all questionnaires and were included in the final analysis (Figure 1). Table 1 summarizes participants’ demographics, sleep profiles, and clinical characteristics. The mean age was 73.4 years (±6.99), with 39 men (43.8%) and 50 women (56.2%). The mean average PSQI global score (6.35 ± 3.84) exceeded the cutoff, whereas mean scores for HAM-A (4.17 ± 3.84), HAM-D (2.53 ± 2.62), AES (25.47 ± 6.85), and TSII (0.52 ± 0.78) all fell within normal ranges.

**Table 1:** Demographic and clinical characteristics of 89 participants.

Characteristics	Values
Age, mean ± S.D. (years)	73.35 ± 6.99
Sex	
Female *n* (%)	50 (56.2)
Male *n* (%)	39 (43.8)
Education in years, mean (%)	
High school graduate or less	28 (31.5)
Junior college	26 (29.2)
College	35 (39.3)
Polysomnographic measures	
Total sleep time, min ± S.D.	359.31 ± 85.13
Sleep efficiency, % ± S.D.	77.12 ± 13.62
Sleep onset latency, min ± S.D.	22.02 ± 22.53
Wake after sleep onset, min ± S.D.	87.11 ± 64.91
N1 sleep duration, % ± S.D.	23.36 ± 9.91
N2 sleep duration, % ± S.D.	54.92 ± 9.50
N3 sleep duration, % ± S.D.	3.70 ± 5.41
Rapid eye movement sleep, % ± S.D.	17.99 ± 5.15
Arousal index, events/h ± S.D.	26.97 ± 11.59
PSQI scores ±S.D.	
PSQI Sleep Quality	1.44 ± 0.82
PSQI Sleep Latency	1.08 ± 1.04
PSQI Sleep Duration	0.93 ± 0.97
PSQI Sleep Efficiency	0.56 ± 0.96
PSQI Sleep Disturbances	1.13 ± 0.43
PSQI use of sleeping medication	0.98 ± 1.34
PSQI Daytime Dysfunction	0.22 ± 0.44
PSQI total	6.35 ± 3.84
Psychological and Cognitive measures, total scores ± S.D.	
HAM-A	4.17 ± 3.84
HAM-D	2.53 ± 2.62
AES	25.47 ± 6.85
TSII	0.52 ± 0.78
UCLALS	26.91 ± 7.72
ECog Total	40.17 ± 1.96

PSQI, Pittsburgh Sleep Quality Index; HAM-A, Hamilton Rating Scale for Anxiety; HAM-D, Hamilton Rating Scale for Depression; AES, Apathy Evaluation Scale; TSII, Triggers of Suicidal Ideation Inventory; UCLALS, University of California Los Angeles Loneliness Scale; ECog, Everyday Cognition scale. Data are presented as mean ± S. D. or n (%).

### Correlation analysis among the subscales of PSQI and PSG indices

The analysis of the correlation between PSQI subscales and PSG indices revealed significant associations, underscoring links between self-reported and objective sleep measures (Table 2). PSQI sleep quality was significantly correlated with PSG WASO (rs = 0.272, *p* = 0.010). PSQI sleep latency was associated with PSG sleep efficiency; (rs = −0.258, *p* = 0.015), SoL; (rs = 0.285, *p* = 0.007), and WASO (rs = 0.285, *p* = 0.007). PSQI sleep disturbances were correlated with PSG SE (rs = −0.285, *p* = 0.007) and WASO (rs = 0.255, *p* = 0.016). PSQI use of sleeping medication was correlated with PSG N2 sleep duration (rs = 0.363, *p* < 0.001), N3 sleep duration (rs = −0.308, *p* = 0.003), and REM sleep (rs = −0.336, *p* = 0.001). Finally, PSQI global scores correlated with PSG WASO (rs = 0.216, *p* = 0.042) and N2 sleep duration (rs = 0.225, *p* = 0.034).

**Table 2:** Correlation analysis among indices of PSQI and PSG.

Indices of PSG	PSQI sleep quality	PSQI sleep latency	PSQI sleep duration	PSQI sleep efficiency	PSQI sleep disturbances	PSQI med	PSQI daytime dysfunction	PSQI total
TST	0.029	−0.003	−0.096	−0.057	−0.125	−0.033	−0.081	−0.032
SE	−0.174	−0.258*	0.087	−0.154	−0.285**	−0.051	−0.104	−0.170
SoL	0.032	0.285**	−0.103	0.176	0.176	0.197	0.103	0.201
WASO	0.272**	0.285**	−0.053	0.112	0.255*	0.053	0.170	0.216*
N1 sleep duration	−0.034	−0.012	−0.127	0.045	0.120	−0.081	−0.098	−0.062
N2 sleep duration	0.091	0.092	0.063	0.040	−0.118	0.363**	0.064	0.225*
N3 sleep duration	−0.031	−0.011	−0.020	−0.016	0.044	−0.308**	0.225*	−0.133
REM	−0.057	−0.116	0.081	−0.023	−0.042	−0.336**	−0.055	−0.177
Arousal Index	0.022	0.040	−0.085	0.111	0.081	−0.126	−0.099	−0.048

PSQI Med, Pittsburgh Sleep Quality Index use of sleeping medication; TST, total sleep time; SE, sleep efficiency; SoL, sleep onset latency; WASO, wake after sleep onset. Bold, statistically significant. *** *p*-value <0.001 (2-tailed); ** *p*-value < 0.01 (2-tailed); * *p*-value < 0.05 (2-tailed).

### Key indicators among PSG indices, psychological distress, loneliness, and cognitive functioning are associated with poor subjective sleep

Logistic regression analysis defined poor sleep status according to PSQI scores. In Model 1, PSG SE, SoL, and WASO were significantly associated with poor sleep. After adjusting for psychological distress and loneliness in Model 2, PSG SE, SoL, WASO, and depression remained significant. In Model 3, which additionally adjusted for cognitive function, the same variables—PSG SE, SoL, WASO, and depression—remained significantly associated with poor sleep. The results are listed in Table 3.

**Table 3:** Logistic regression analysis of the factors contributing to subjective poor sleep.

Subjective poor sleep determined using PSQI						
Variables	**Model 1 (*n* = 89)**		**Model 2 (*n* = 89)**		**Model 3 (*n* = 89)**	
	OR (95% CI)	*p*-value	OR (95% CI)	*p*-value	OR (95% CI)	*p*-value
Sex^a^	0.964 (0.219–4.235)	0.961	0.302 (0.029–3.176)	0.319	0.285 (0.025–3.204)	0.309
Age	1.025 (0.922–1.14)	0.635	1.117 (0.936–1.332)	0.217	1.127 (0.940–1.351)	0.198
Education	0.786 (0.343–1.802)	0.570	1.154 (0.37–3.598)	0.805	1.114 (0.350–3.543)	0.855
TST	0.988 (0.973–1.004)	0.164	0.983 (0.962–1.004)	0.130	0.984 (0.962–1.006)	0.142
SE	1.579 (1.063–2.347)	**0.024**	2.348 (1.114–4.949)	**0.025**	2.414 (1.124–5.186)	**0.024**
SoL	1.108 (1.021–1.203)	**0.014**	1.192 (1.028–1.382)	**0.020**	1.201 (1.033–1.396)	**0.017**
WASO	1.082 (1.011–1.159)	**0.021**	1.15 (1.012–1.306)	**0.030**	1.157 (1.015–1.319)	**0.030**
N1 sleep duration	0.316 (0.014–6.932)	0.465	1.429 (0.012–169.509)	0.883	1.117 (0.008–148.587)	0.965
N2 sleep duration	116.377 (0.068–1.989E+5)	0.210	135.986 (0.004–4.257E+6)	0.352	94.454 (0.002–3.631E+6)	0.398
N3 sleep duration	1.036 (0.571–1.878)	0.907	1.289 (0.533–3.116)	0.578	1.286 (0.531–3.112)	0.578
REM	0.878 (0.75–1.029)	0.111	0.896 (0.735–1.093)	0.282	0.892 (0.730–1.090)	0.264
Arousal Index	1.099 (0.973–1.24)	0.126	1.048 (0.903–1.217)	0.531	1.039 (0.891–1.211)	0.625
HAM-A			0.607 (0.283–1.301)	0.200	0.608 (0.278–1.329)	0.212
HAM-D			3.169 (1.083–9.268)	**0.035**	3.355 (1.094–10.293)	**0.034**
AES			0.966 (0.795–1.173)	0.728	0.967 (0.792–1.179)	0.739
TSII			7.519 (0.973–58.092)	0.053	8.215 (1.001–67.393)	0.050
UCLALS			0.928 (0.81–1.062)	0.280	0.918 (0.798–1.056)	0.232
ECog Total				0.789	0.801 (0.533–1.205)	0.288

TST, total sleep time; SE, sleep efficiency; SoL, sleep onset latency; WASO, wake after sleep onset; HAM-A, Hamilton Rating Scale for Anxiety; HAM-D, Hamilton Rating Scale for Depression; AES, Apathy Evaluation Scale; TSII, Triggers of Suicidal Ideation Inventory; UCLALS, University of California Los Angeles Loneliness Scale; ECog, Everyday Cognition scale. Subjective poor sleep and non-subjective poor sleep were the dependent variables. Model 1 was adjusted for sex, age, and education. Model 2 was adjusted for Model 1, as well as psychological distress (HAM-A, HAM-D, AES, TSII) and loneliness (UCLALS). Model 3 was adjusted for Model 2, as well as cognitive functioning (ECog scales). ^a^Men vs women; Bold, statistically significant; OR: odds ratio, CI: confidence interval.

### Stepwise regression model for PSG SE, SoL, WASO, and depression

Building on the dominant indicators identified above, stepwise regression models further clarified indices of subjective sleep condition. Poor sleep is defined by the PSQI dichotomous method. Education, age, PSQI disturbance, and sex were significantly associated with PSG SE, explaining 13.4, 7.5, 7.2, and 4.8% of its variance, respectively. Education and PSQI latency were significantly associated with PSG SoL, explaining 13.4 and 8.7% of the variance, respectively. PSQI quality, education, and sex were significantly associated with PSG WASO, explaining 9.3, 5.5, and 5.1% of the variance, respectively. Finally, PSQI latency and PSQI quality were significantly associated with depression, explaining 20.9 and 4.2% of the variance, respectively. Results are summarized in Table 4.

**Table 4:** Independent factors associated with sleep efficiency and wake after sleep onset of PSG.

Dependent variable	Independent variables	B	95% CI	*t*	*R^2^* change	*p*-value
PSG SE	Education	5.167	2.126–8.207	3.379	0.134	0.001
	Age	−0.490	−0.849–−0.132	−2.722	0.075	0.008
	PSQI Disturbance	−8.637	−14.300–−2.975	−3.033	0.072	0.003
	Sex	−6.118	−11.105–−1.131	−2.440	0.048	0.017
PSG SoL	Education	−8.616	−13.734–-3.498	−3.346	0.134	0.001
	PSQI Latency	6.423	2.305–10.542	3.101	0.087	0.003
PSG WASO	PSQI Quality	21.290	6.099–36.480	2.787	0.093	0.007
	Education	−23.183	−38.330–−8.036	−3.043	0.065	0.003
	Sex	29.998	4.597–55.398	2.348	0.051	0.021
Depression	PSQI Latency	0.805	0.248–1.362	2.874	0.209	0.005
	PSQI Quality	0.779	0.072–1.485	2.191	0.042	0.031

PSG, Polysomnography; SE, sleep efficiency; SoL, sleep onset latency; WASO, wake after sleep onset. Independent variables included three demographic variables (sex, age, and education) and all subscales of PSQI (sleep quality, latency, duration, efficiency, disturbances, use of sleeping medication, and daytime dysfunction).

## Discussion

This study examined associations between specific PSQI components and objective PSG measures, as well as psychological distress, loneliness, and cognitive functioning in healthy older adults. Significant correlations were observed between several PSQI components and PSG metrics. Subjectively poorer sleep quality, longer sleep latency, greater sleep disturbance, and poorer overall sleep outcomes were associated with PSG SE, SoL, and WASO. These results support previous evidence that poor self-reported sleep quality is linked to objective WASO (10). Similarly, a prior study using the Insomnia Severity Index found that self-reported difficulty maintaining sleep correlated with PSG-measured WASO (29). In this study, increased use of sleep medication was associated with longer N2 sleep and shorter N3 and REM stages. Hypnotics are frequently prescribed to older adults as a first-line treatment for insomnia (30). Benzodiazepines (BZDs) and non-BZDs may contribute to this pattern, as they are known to increase stage 2 of NREM sleep, while BZDs also reduce slow-wave sleep (stages 3 and 4) and decrease REM duration (31, 32). Consistent with earlier findings, global PSQI scores did not consistently align with objective sleep measures (13, 33). Specifically, PSQI-reported sleep duration and efficiency did not correspond with objective TST and SE, underscoring the persistent discrepancies between subjective perceptions and objective assessments.

Previous studies have shown that individuals with psychosomatic disorders—commonly identified as poor sleepers based on elevated PSQI scores—display objectively lower SE and longer WASO compared to those with lower PSQI scores, who generally report better sleep quality (10). Although our participants were cognitively healthy older adults, our findings reinforce the importance of objective SE and WASO as indicators reflecting subjective sleep quality assessed by the PSQI. Age-related sleep changes are well established: older adults, irrespective of sex, typically experience shorter total sleep, lower SE, and more frequent nocturnal arousals. However, sex-specific patterns have also been reported, with older women more likely to present prolonged sleep latency, frequent nocturnal awakenings, and earlier-than-desired waking times (34). Discrepancies between subjective and objective sleep measures have been further documented. For instance, individuals with insomnia complaints often report markedly impaired perceived SE, latency, WASO, and TST compared to that of controls. Notably, subjective insomnia reports are more closely associated with increased WASO than with prolonged sleep latency (35). In this context, WASO emerges as a critical factor. One study demonstrated that elevated WASO episodes were significantly associated with non-robust or frail physical status in older adults (36). Thus, WASO is linked to subjective sleep quality and physiological vulnerability.

Our findings indicate that depression is associated with subjective sleep latency and quality. This aligns with earlier research showing that poor sleep quality, prolonged SoL, and reduced sleep duration are linked to heightened symptoms of depression and anxiety (37, 38). Additionally, one study identified a stronger predisposition toward depression as a key predictor of poor self-reported sleep quality (38). Supporting this pattern, a large community-based study found that nearly half of older adults experienced poor sleep, which was strongly associated with elevated depressive and anxiety symptoms (39). Consistently, another study demonstrated independent associations between depression, anxiety, and sleep-related complaints in Asian older adults, underscoring the bidirectional relationship between sleep and emotional dysregulation (40). Collectively, our findings indicate that psychological distress—particularly depressive symptoms—may serve as a critical confounder in the relationship between subjective sleep complaints and objective sleep characteristics.

Our results further demonstrate that among PSQI components, specifically sleep latency and quality, the objective indices most closely related to subjective complaints are SoL and WASO. These findings underscore the critical value of the two objective measures and further highlight the discrepancies observed between subjective and objective assessments of sleep.

Regarding cognitive functioning, Tang et al. reported, contrary to their initial hypothesis, that objective sleep parameters were not significantly associated with cognitive decline over time (41). Our findings are consistent with this observation. However, other studies have shown that poorer global sleep quality is linked to lower baseline executive function and steeper cognitive decline over time (42). Among cognitively healthy older adults, the relationship between sleep and cognition remains inconclusive and warrants further investigation with larger, longitudinal datasets.

### Study strengths and limitations

This study has limitations. First, the relatively small sample size (n = 89) may restrict the generalizability of the results to broader populations. The results should be interpreted cautiously, and larger cohorts are warranted to validate and extend these findings. Second, the cross-sectional design precludes establishing causal relationships between PSQI components, PSG metrics, and outcomes related to psychological distress, loneliness, and cognitive functioning. Longitudinal studies are required to clarify the temporal dynamics among these variables. Third, reliance on self-reported measures, including the PSQI and questionnaires for psychological distress, loneliness, and cognition, may introduce reporting bias and affect measurement accuracy. Besides, only one night of home-based PSG was recorded. The potential impact of first-night effects cannot be fully excluded. Although home-based assessment may reduce unfamiliarity-related disturbance, two-night recordings are recommended for more stable estimates of sleep architecture in the future. Finally, although age and sex were considered, other potential confounders, such as socioeconomic status, medication use, and comorbidities, should also be incorporated into future analyses.

## Conclusion

In conclusion, specific PSQI subscales—particularly sleep quality, latency, and disturbance—were significantly associated with key PSG parameters, including SE, SoL, and WASO. Further analyses identified PSG SE, SoL, WASO, and depression as the strongest influencing indices of subjective poor sleep. Additionally, certain PSQI subscales—sleep quality, latency, and disturbance—showed significant correlations with key PSG parameters and depression. These findings emphasize the partial convergence between subjective and objective sleep measures and highlight the additional influence of emotional and demographic factors on perceived sleep quality in healthy older adults. Given the high prevalence of sleep problems and their emotional correlates among community-dwelling older adults (39), our study provides preliminary yet important evidence supporting the integration of subjective and objective sleep assessments with emotional and cognitive indicators as part of an early and precise evaluation framework for personalized healthy aging. Future research should employ longitudinal designs with larger and more diverse samples to clarify these relationships and strengthen their implications for clinical practice.

## Data Availability

The original contributions presented in the study are included in the article/supplementary material, further inquiries can be directed to the corresponding author.
